# The Psychological and Relational Impact of Mayer‐Rokitansky‐Kuster‐Hauser Syndrome: Perspectives of Patients, Mothers and Partners

**DOI:** 10.1111/ajo.70213

**Published:** 2026-11-13

**Authors:** Kuangjun Li, Claire Elizabeth Wakefield, Brittany Claire McGill, Rebecca Deans

**Affiliations:** ^1^ School of Clinical Medicine, UNSW Medicine & Health, Discipline of Women's Health UNSW Sydney Randwick New South Wales Australia; ^2^ Behavioural Sciences Unit, School of Clinical Medicine, UNSW Medicine & Health, Discipline of Paediatrics and Child Health UNSW Sydney Randwick New South Wales Australia; ^3^ General Medicine Department The Children's Hospital at Westmead Westmead New South Wales Australia; ^4^ Division of Quality of Life and Pediatric Palliative Care, Department of Pediatrics Stanford University and Stanford Medicine Children's Health Palo Alto California USA; ^5^ The Royal Hospital for Women Randwick New South Wales Australia

## Abstract

**Background:**

Mayer‐Rokitansky‐Küster‐Hauser syndrome (MRKHS) is a rare reproductive disorder with long‐term psychosocial impacts. However, understanding of family and partner relationships that integrates the perspectives of patients, parents, and partners remains limited.

**Aims:**

To evaluate the psychosocial impact of MRKHS with a focus on psychological and relational functioning, from patient, parent, and partner perspectives in an Australian cohort.

**Materials and Methods:**

Patients with MRKHS aged ≥ 18 years (*n* = 23), their parents (*n* = 8), and partners (*n* = 3) were recruited from a tertiary centre in Sydney, Australia. Three tailored questionnaires used validated self‐report measures to assess physical and psychological health, family functioning, relationship satisfaction, fertility‐related quality of life (QoL), sexual function, and personal identity. Comparison data were drawn from published validation studies.

**Results:**

Patients with MRKHS reported comparable self‐perceived health and relationship satisfaction to reference groups, but higher anxiety (*p* = 0.002), more significant dyspareunia (*p* = 0.006), and poorer perceived family functioning (*p* < 0.001). They reported better overall fertility‐related QoL than couples undergoing fertility treatment (*p* = 0.03) but had lower scores on the relationship subscale (*p* = 0.002). MRKHS had a central impact shaping self‐identity (*p* = 0.002). Mothers and partners reported positive wellbeing and family or relationship functioning, although the findings are preliminary due to the limited sample size.

**Conclusion:**

MRKHS affects multiple facets of patients' lives, particularly psychological well‐being, pain‐related sexual function, family dynamics, and personal identity. Optimal care should incorporate tailored psychological support and facilitate open communication between patients and their support network. Larger studies are warranted to examine the psychological impact of MRKHS on parents and partners.

## 1 Introduction

Meyer‐Rokitansky‐Küster‐Hauser syndrome (MRKHS) is a rare congenital condition that affects approximately 1 in every 5000 females [1]. It is characterised by aplasia or hypoplasia of Müllerian duct‐derived organs such as the uterus, fallopian tubes, and upper two‐thirds of the vagina. Patients with MRKHS have otherwise normal female secondary sexual characteristics and external genitalia. MRKHS is typically diagnosed during adolescence, with primary amenorrhea as the most common presenting complaint. Other manifestations of MRKHS include dyspareunia, apareunia, and infertility. The primary treatment involves the creation of a neovagina through non‐surgical vaginal dilation or surgical vaginoplasty to facilitate penetrative sexual intercourse. The recent emergence of uterine transplantation has allowed individuals with MRKHS to achieve genetic and gestational parenthood [1].

Psychological wellbeing of patients with MRKHS has been widely studied [2, 3, 4]. A 2022 systematic review comparing psychological wellbeing, quality of life, and sexual function in individuals with MRKHS with those without the condition found that the MRKHS cohort had higher rates of anxiety and depression symptoms, lower mental health related quality of life, and poorer sexual function outcomes [4]. Notably, patients identified challenges navigating mother–child dynamics, intimacy and self‐identity as recurring and inter‐related themes in a qualitative study exploring the psychological impact of MRKHS [5]. However, there remains limited understanding regarding these relational and identity aspects of the MRKHS experience in the current literature [1, 6]. Parents and partners have been identified as key sources of support for individuals with MRKHS in previous studies [2, 5]. However, the perspectives of parents and partners have largely been understudied in the current literature.

The primary objective of this study was therefore to examine the psychosocial impact of MRKHS in an Australian cohort, from the perspectives of patients, their parents and partners, using a series of validated self‐report measures. We aimed to assess physical and psychological wellbeing, family functioning, relationship satisfaction, fertility‐related QoL, sexual function, and personal identity.

## 2 Materials and Methods

### 2.1 Settings

This cohort study was conducted at the Royal Hospital for Women (RHW) in Sydney, a tertiary referral hospital specialising in women's health in Australia. The RHW has a long‐standing history of caring for patients with MRKHS and hosts annual MRKHS support groups for patients and their families.

### 2.2 Ethics

Ethics approval was granted by the South Eastern Sydney Local Health District Human Research Ethics Committee (HREC ref. no: 14/269). Informed consent was obtained in accordance with the Privacy Commissioner's Guidelines for Research and the Health Records and Information Privacy Act 2002 (NSW).

### 2.3 Design

We identified patients with MRKHS who were registered in the RHW clinical database between 2015 and 2016. Patients under the age of 18 years, or those unable to provide informed consent due to language barriers or intellectual disability, were excluded.

An invitation package containing study information was emailed to eligible patients, inviting participation from themselves, their parents, and their partners (if applicable). Additionally, invitation packages were distributed at two MRKHS support group meetings held at the RHW. Interested participants could email the research team to request group‐specific, password‐protected links to the online questionnaire. Informed consent was implied through completion of the questionnaire. One reminder email was sent to non‐respondents.

The study comprised three tailored online questionnaires, targeted respectively at patients with MRKHS, their parents, and their partners. The questionnaires were designed to collect information on basic demographics, clinical background, and the psychosocial impact of MRKHS across several domains: physical and psychological wellbeing (EQ‐5D‐5L [7]; Depression Anxiety and Stress Scale [DASS‐21] [8]; Rosenberg Self‐Esteem Scale [RSES] [9]; Emotion Thermometers [ET] [10]), sexual function (Female Sexual Function Index [FSFI] [11]), fertility‐related QoL (Fertility Quality of Life [FertiQoL] [12]), family and partner relationships (Family Assessment Device [FAD] [13]; Relationship Assessment Scale [RAS] [14]), and self‐identity (Centrality of Event Scale [CES] [15]) (see Supplementary Appendix A).

### 2.4 Statistical Analysis

Each participant was assigned an individualised confidential identifier code, designed to enable intra‐group analysis and linkage of patient–parent–partner triads for correlation analysis.

Statistical analysis was performed using IBM SPSS 28.0 (IBM Corp, Chicago, IL, USA). Sample responses were scored according to the instructions and compared with established cutoff scores from the respective validated self‐report scales. Sample means were calculated and compared with published comparison group means using the one‐sample *t*‐ test. Categorical data were analysed using the one‐sample chi‐square test to compare the observed distribution with frequencies from published comparison data. Comparison groups were sourced from the validation studies for each scale, prioritising national normative data (EQ‐5D‐5L, DASS‐21, RSES, FAD) or gender‐matched normative samples (ET, FSFI, RAS), followed by samples from the original validation studies (FertiQoL, CES) (Supplementary Appendix A). A *p* value of < 0.05 was considered statistically significant.

## 3 Results

A total of 23 patients, 8 parents and 3 partners were included in the study (Figure 1). This included 5 patient‐parent pairs, 2 patient‐partner pairs and 1 patient‐parent‐partner triad. Table 1 summarises their demographic information. The median age of patients was 27 years (IQR 13). Forty‐three percent of patients reported a previous mental health diagnosis. Notably, only female parents and male partners participated in the study. Due to the small number of intra‐group linkages, statistical analyses for correlations between participant groups were not performed.

**FIGURE 1:**
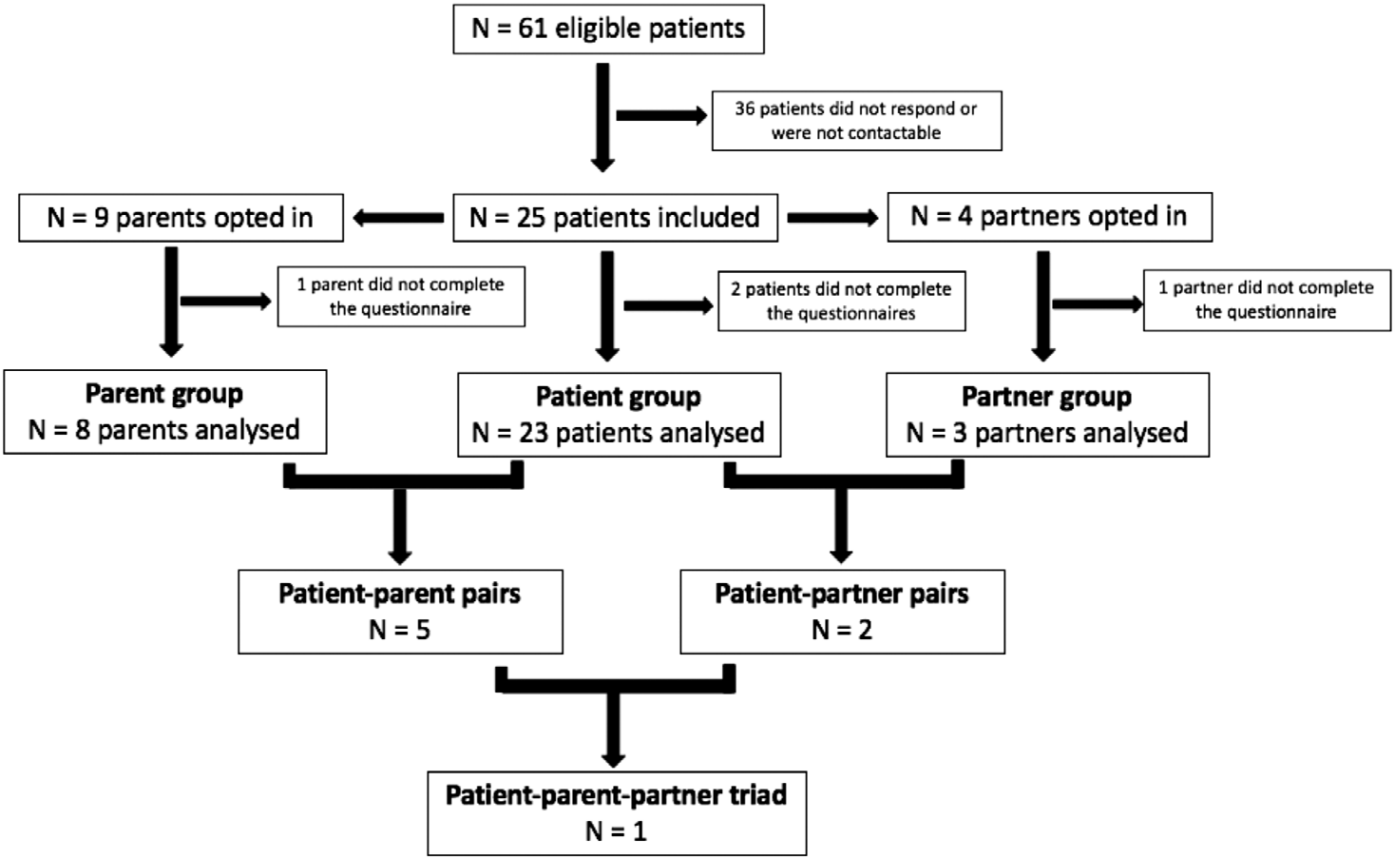
Participant flow chart.

**TABLE 1:** Participant demographics.

Characteristics, *n* (%)	Patient group (*n* = 23)	Parent group (*n* = 8)	Partner group (*n* = 3)
Sex			
Female	23 (100)	8 (100)	0
Male	0	0	3 (100)
Median age, years (IQR)	27 (13)	63 (9)	44 (9)
Education			
Tertiary education and above	20 (87)	3 (38)	1 (33)
Secondary education only	3 (13)	5 (62)	2 (67)
Employment			
Student	4 (18)	0	0
Full‐time employment	15 (65)	5 (63)	3 (100)
Pensioner	4 (18)	3 (37)	0
Relationship status			
Single	10 (43)	0	n.a.
De‐facto/married	10 (43)	6 (75)	3 (100)
Separated/divorced	3 (14)	2 (25)	n.a.
Previous mental health diagnosis			
Depression	6 (26)	2 (25)	1 (33)
Anxiety	4 (17)	1 (13)	0
Children			
Yes	1^a^ (4)	n.a.	1^b^ (33)
No	22 (96)	n.a.	2 (66)
Sexual orientation			
Heterosexual	14 (82)	n.a.	3 (100)
Homosexual	0	n.a.	0
Bisexual	2 (12)	n.a.	0
Asexual	1 (4)	n.a.	0
Sexually active			
Yes	19 (83)	n.a.	3 (100)
Prefer not to answer	4 (17)	n.a.	0

Abbreviation: n.a., not applicable or not asked.

^a^ The only patient who reported having a child indicated this was through adoption.

^b^ One male partner reported having children from a previous relationship.

Patients' clinical characteristics are shown in Table 2. Over half of the patient cohort (56%) reported at least one physical anomaly commonly associated with MRKHS. Regarding treatment, nearly half of the group (43%) received non‐surgical vaginal dilation, while 35% reported no previous medical treatment. Psychological counselling had been accessed by 30% of patients.

**TABLE 2:** Patient clinical characteristics.

MRKHS diagnosis	Patient group (*n* = 23)
Age of diagnosis, median years (IQR)	16 (2)
Duration since diagnosis, median years (IQR)	10 (10)
Associated physical anomalies, *n* (%)	
Renal	4 (17)
Skeletal	6 (26)
Hearing	2 (9)
Cardiac	1 (4)
Treatment, *n* (%)	
Surgical vaginoplasty	3 (13)
Non‐surgical dilation therapy at home	5 (22)
Non‐surgical dilation therapy in hospital	7 (30)
No treatments	8 (35)
Psychological therapy, *n* (%)	7 (30)

Table 3A presents the psychosocial outcomes for the patient group compared to the respective comparison groups, and response frequencies relative to the cutoff scores where applicable. On the EQ‐5D‐5L scale, patients with MRKHS reported comparable health‐related QoL to the Australian national average (mean utility score = 0.91 vs. 0.86), suggesting generally positive self‐perceived health [16].

**TABLE 3A:** Self‐rated quality of life outcomes in the patient group vs. comparison groups.

Psychological Scale	Patient group^a^	Comparison	*p*	%^b^
EQ‐5D‐5L				
Utility score, mean (SD)	0.91 (14.3)	0.86 (0.19)	0.094	n.a.
EQ VAS, mean (SD)	71.8 (22.6)	73.2 (21.7)	0.77	n.a
DASS‐21				
Depression, mean (SD)	3.91 (4.45)	2.57 (3.86)	0.16	26
Anxiety, mean (SD)	3 (4.08)	1.74 (2.78)	0.15	30
Stress, mean (SD)	5.87 (5.29)	3.99 (4.24)	0.10	30
Rosenberg Self‐Esteem Scale				
Mean (SD)	29.95 (6.27)	31.07 (5.15)	0.40	74
Emotional Thermometer^c^				
Distress (%)	30.4	42.2	0.25	30.4
Anxiety (%)	48	15.7	0.002	48
Depression (%)	30.4	19.3	0.18	30.4
Anger (%)	30.4	25.3	0.57	30.4
Need help (%)	30.4	13.9	0.08	30.4
Centrality of Event Scale (*n* = 17)				
Mean (SD)	4.23 (0.77)	3.56 (0.8)	0.002	n.a.
Fertility Quality of Life				
Emotion, mean (SD)	59.96 (27.0)	45.1 (23.2)	0.02	n.a.
Mind/body, mean (SD)	73.37 (24.3)	54.86 (21.2)	0.001	n.a.
Relation, mean (SD) (*n* = 17)	57.25 (16.29)	68.7 (19.2)	0.002	n.a.
Social, mean (SD)	59.6 (18.9)	51.1 (20.6)	0.04	n.a.
Core FertiQoL, mean (SD)	62.6 (16.7)	54.6 (16.8)	0.03	n.a.
Female Sexual Function Index (*n* = 19)				
Desire, mean (SD)	4.42 (0.98)	4.28 (1.12)	0.54	n.a.
Arousal, mean (SD)	4.80 (1.35)	5.08 (1.11)	0.38	n.a.
Lubrication, mean (SD)	4.88 (1.52)	5.45 (1.14)	0.12	n.a.
Orgasm, mean (SD)	4.78 (1.22)	5.05 (1.3)	0.35	n.a.
Satisfaction, mean (SD)	4.80 (1.42)	4.23 (1.2)	0.1	n.a.
Pain, mean (SD)	4.21 (1.83)	5.51 (1.29)	0.006	n.a.
Total, mean (SD)	27.89 (6.38)	30.75 (4.8)	0.07	68
Family Assessment Device				
Mean	2.21	1.63	< 0.001	52
Relationship Assessment Scale (*n* = 11)	4.46 (0.47)	4.33 (0.63)	0.41	80

Abbreviation: n.a., not applicable.

^a^ *n* = 23 unless otherwise specified.

^b^ Proportion of participants scoring above clinical cutoff scores.

^c^ Percentage of patients self‐rating ≥ 4, indicating clinically relevant emotional distress.

On the Depression Anxiety and Stress Scale (DASS‐21), the MRKHS group did not differ significantly from a national adult population sample on any of the three subscales, including depression, anxiety, and stress [17]. Notably, close to one third patients (26%–30%) reported emotional distress above the respective cutoff scores on each of the three domains.

On the Emotion Thermometers (ET), almost half of the MRKHS patient group (48%) reported anxiety‐related distress, using the cutoff score of ≥ 4. Additionally, the reported anxiety level was significantly higher compared to a normative German female sample (*p* = 0.002) [18]. No significant differences were observed between the MRKHS group and the comparison group across all other subscales.

On the Rosenberg Self‐Esteem Scale (RSES), there were no significant differences in mean scores between patients with MRKHS and a comparison group of Australian adults (mean = 29.95 vs. 31.07) [19]. Additionally, 74% of patients scored above the theoretical midpoint of 25, suggesting generally positive self‐evaluation among the MRKHS group.

On the Relationship Assessment Scale (RAS), the 10 MRKHS patients who were in a partnered relationship reported a mean score of 4.46, with 8 participants scoring above the cutoff score of 4, suggesting that most perceived their partnerships as positive. The MRKHS group also demonstrated a mean score that is comparable to women in relationships in the original validation study (mean = 4.46 vs. 4.33) [20].

On the Centrality of Event Scale (CES), patients with MRKHS reported significantly higher mean scores compared to the original validation sample of North American undergraduate students (mean = 4.23 vs. 3.56, *p* = 0.002) [15]. This significant difference suggests that the experience of MRKHS plays a central role in shaping patients' self‐identity and life narrative.

In comparison to men and women experiencing fertility issues in the validation study, patients with MRKHS showed significantly better fertility‐related QoL on the total Fertility Quality of Life (FertiQoL) (*p* = 0.03) and subscales including emotional (*p* = 0.02), body (*p* = 0.001), and social (*p* = 0.04) [12]. However, patients with MRKHS scored poorer in the relational subscale (*p* = 0.002), suggesting greater difficulty in partnered relationships associated with infertility.

On the Female Sexual Function Index (FSFI), sexually active participants (*n* = 19) reported comparable results to females without sexual dysfunction in the subscales of desire, arousal, lubrication, orgasm, and satisfaction [21]. Pain was the only functional domain in which MRKHS participants scored significantly lower (*p* = 0.006), suggesting higher levels of pain. The difference in total FSFI score between the MRKHS group and the validation study comparison group was not statistically significant (*p* = 0.07), suggesting that individuals with MRKHS who receive appropriate care may have sexual functioning comparable to that of the general population [21].

Patients with MRKHS had a significantly higher mean score on the Family Assessment Device (FAD) compared to the comparison group of Western Australians (*p* < 0.001), indicating significantly poorer perceived family functioning [22]. Additionally, over half of the MRKHS group (52%) scored above the clinical threshold of 2.0, highlighting challenges in family relationships.

### 3.1 Parent and Partner Group

Summaries of psychosocial outcomes for parents and partners of individuals with MRKHS are presented in Table 3B and Table 3C respectively. Given the small sample size (8 parents and 3 partners), only descriptive statistics were analysed and presented.

**TABLE 3B:** Self‐rated quality of life outcomes in the parent group vs. comparison groups.

Psychological Scale	Parent group^a^	Comparison	%^b^
EQ‐5D‐5L			
Utility score, mean (SD)	0.82 (0.26)	0.86 (0.19)	n.a.
EQ VAS, mean (SD)	70 (16.25)	73.2 (21.7)	n.a
DASS‐21			
Depression, mean (SD)	3.75 (4.59)	2.57 (3.86)	25
Anxiety, mean (SD)	3.75 (3.33)	1.74 (2.78)	37.5
Stress, mean (SD)	5.5 (3.42)	3.99 (4.24)	12.5
Rosenberg Self‐Esteem Scale			
Mean (SD)	30.5 (6.57)	31.07 (5.15)	75
Emotional Thermometer^c^ (*n* = 7)			
Distress (%)	29	42.2	29
Anxiety (%)	29	15.7	29
Depression (%)	29	19.3	29
Anger (%)	14	25.3	14
Need help (%)	14	13.9	14
Family Function Assessment			
Mean	1.63	1.63	12.5

Abbreviation: n.a., not applicable.

^a^ *n* = 8 unless otherwise specified.

^b^ Proportion of participants scoring above clinical cutoff scores.

^c^ Percentage of patients self‐rating ≥ 4, indicating clinically relevant emotional distress.

**TABLE 3C:** Self‐rated quality of life outcomes in the partner group vs. comparison groups.

Psychological Scale	Partner group^a^	Comparison	%^b^
EQ‐5D‐5L			
Utility score, mean (SD)	0.92 (0.05)	0.86 (0.19)	n.a
EQ VAS, mean (SD)	88.3 (7.64)	73.2 (21.7)	n.a
DASS‐21			
Depression, mean (SD)	1.33 (0.33)	2.57 (3.86)	0
Anxiety, mean (SD)	0.33 (0.58)	1.74 (2.78)	0
Stress, mean (SD)	2.33 (2.08)	3.99 (4.24)	0
Rosenberg Self‐Esteem Scale			
Mean (SD)	31.33 (6.35)	31.07 (5.15)	66.7
Emotional Thermometer^c^			
Distress (%)	0	34.9	0
Anxiety (%)	0	8	0
Depression (%)	0	12	0
Anger (%)	66.7	23.6	66.7
Need help (%)	0	6.6	0
Relationship Assessment Scale	4.62 (0.08)	4.30 (0.64)	100

Abbreviation: n.a., not applicable.

^a^ *n* = 3 unless otherwise specified.

^b^ Proportion of participants scoring above clinical cutoff scores.

^c^ Percentage of patients self‐rating ≥ 4, indicating clinically relevant emotional distress.

The parent group's scores were comparable to the population norms reported in the validation studies, suggesting generally positive self‐perceived health on the EQ‐5D‐5L, satisfactory self‐esteem on the RSES, and comparable family functioning [16, 19, 22]. Clinically relevant depression and anxiety above the established cutoff scores were identified in nearly a third of the parent group on the DASS‐21 and ET.

Among the partner group, all three participants reported consistently high self‐perceived health, self‐esteem, and relationship function on the EQ‐5D‐5L, RSES, and RAS respectively. On the DASS‐2, all partners scored below the cutoff scores across all three emotional distress subscales, indicating no clinically significant depression, anxiety or stress.

## 4 Discussion

This study provides a multi‐perspective assessment of the psychosocial impact of MRKH syndrome. In a cohort of Australian patients, parameters not widely understood in the MRKHS population, including fertility‐related QoL, family functioning, and relationship satisfaction, were assessed, strengthening our understanding of the experiences and impact of MRKHS on individuals and their families.

Across the patient, parent, and partner groups, self‐perceived health and self‐esteem reported on the EQ‐5D‐5L and RSES were comparable to those of the representative samples of the Australian adult population [16, 19]. In terms of psychological health, clinically relevant emotional distress was reported by one third to one half of individuals with MRKHS. However, these findings were not consistently statistically significant when compared with unaffected individuals, likely due in part to our limited sample size. Our findings are consistent with a 2022 systematic review, which reported that patients with MRKHS more commonly experience mental health symptoms without apparent impact on physical health related quality of life [3, 4]. Additionally, a higher proportion of mothers of patients with MRKHS reported anxiety symptoms compared with the Australian general population (37.5% vs. 26%) [23]. Further research is needed to characterise the nature of anxiety experienced by these mothers and to contextualise it in relation to their daughters' MRKHS diagnosis and impact.

Sexual function in individuals with MRKHS has been widely studied and the results have been highly heterogeneous [2, 4, 24]. In our study, the MRKHS group reported higher levels of pain; however, their overall sexual functioning was comparable to that of the general population. These findings suggest that pain and discomfort during sexual intercourse may not be fully alleviated by neovagina creation alone in individuals with MRKHS, consistent with the findings of a 2022 systematic review [4]. Our findings also highlight that sexual experiences in MRKHS are multi‐factorial and influenced by treatment modality and psychosocial factors [4].

Infertility is recognised as one of the most challenging aspects of MRKH syndrome [5, 25]. Compared to individuals experiencing acquired fertility issues, the MRKHS group reported better fertility‐related quality of life scores. Our cohort's median duration of 10 years since diagnosis likely allowed for a longer period of adjustment. Consistent with a 2024 study using the same psychometric scale, the overall higher fertility‐related QoL in our study likely reflects personal acceptance of the diagnosis and its associated uterine factor infertility [26]. Despite this adjustment, significantly higher scores on the Centrality of Event Scale indicate that MRKHS continues to play a central role in shaping self‐identity and life experiences, even long after diagnosis.

Less than half of our patient cohort (43%) were in a partner relationship at the time of study recruitment. While this is likely multi‐factorial, individuals with MRKHS have previously reported challenges in disclosing their diagnosis and its fertility implications to partners due to fear of relationship breakdown [3, 5, 27]. Furthermore, poorer scores on the Fertility Quality of Life relational subscale were observed among individuals with MRKHS, irrespective of relationship status, suggesting an ongoing influence of infertility on intimate relationships. Despite these challenges, the partnered individuals in our study reported overall satisfactory relationship status on the Relationship Assessment Scale. This finding aligns with previous research showing that individuals with MRKHS tend to have fewer partners, longer relationships, and stronger commitment compared to other sexually active females [28]. Furthermore, our small cohort of partners demonstrated overall satisfactory psychological and relational function, consistent with prior studies [6]. These results suggest that relationship satisfaction is multifaceted and may be independent of sexual function and fertility issues [29].

While some participants viewed family relationships as suboptimal, their mothers perceived them as satisfactory, suggesting differing perspectives within family dynamics and adjustment following diagnosis. Previous qualitative studies have reported that mothers are often deeply emotionally invested in their daughters' condition, sometimes leading consultations with health professionals or sharing the diagnosis with family members without the patient's consent [5, 24]. Conflict around diagnosis disclosure can compromise patient privacy and autonomy, potentially straining the mother–child relationship. These results highlight the importance of providing tailored psychological counselling to mothers to ensure parental support aligns with the patient's wishes, facilitates conversations around diagnosis disclosure, and provides opportunities for debriefing. Our study was unable to recruit fathers, so their perspectives, whilst equally important, are not represented.

The main limitation of the study is its small sample size, which may under‐represent the respective participant groups. For example, with only eight mothers and three male partners, we were unable to draw any firm conclusion regarding the psychosocial and relational impact of MRKHS on these groups. As MRKHS is a rare congenital condition, with few new cases added to the patient register each year, and as most participants were recruited through one of two support group meetings rather than the patient register, recruitment was limited to a 1‐year period and a single tertiary centre. Future studies could benefit from collaborative, multi‐centre, and potentially international recruitment to increase participant numbers and improve statistical power. The low response rate among parent and partner groups may reflect the indirect email recruitment method. Additionally, it is also important to acknowledge that time has lapsed since the commencement of this study. This should be considered when interpreting the findings. Furthermore, the study findings were compared with data from the most comparable published validation study, an approach that has been adopted in previous MRKHS research and provided a pragmatic method of contextualising outcomes across multiple validated measures and participant groups. However, an age and gender matched control group would strengthen future study findings and allow for more robust comparisons. Future research with larger and more diverse samples, longitudinal study designs, and qualitative methods to assess psychosocial variables at key time points, including diagnosis, before and after treatment, and long‐term follow up, would allow for stronger and more generalisable conclusions.

In conclusion, MRKH syndrome is a lifelong congenital condition that impacts multiple facets of life. It appears to negatively affect patients' psychological well‐being, family functioning, and pain‐related aspects of sexual outcomes, and plays a central role in shaping personal identity. However, our findings did not indicate significant differences in self‐perceived health, self‐esteem, overall sexual function, or partner relationship among MRKHS patients. Optimal care should incorporate tailored individual psychological support and facilitate open communication between patients and their support network, including mothers and partners.

## Funding

Claire Wakefield is supported by the National Health and Medical Research Council (APP200300). The remaining authors have nothing to report.

## Conflicts of Interest

The authors declare no conflicts of interest.

## Supporting information


**Appendix A** Psychometric Scales.

## Data Availability

The data that support the findings of this study are available from the corresponding author upon reasonable request.
