# Supplementary material for: The Psychological and Relational Impact of Mayer‐Rokitansky‐Kuster‐Hauser Syndrome: Perspectives of Patients, Mothers and Partners
Source: Aust N Z J Obstet Gynaecol. 2026 Nov 13;66(6):e70213. doi: 10.1111/ajo.70213 (PMC13768570; doi:10.1111/ajo.70213)
Supplement: Supplementary file 1 [file AJO-66-0-s001.docx]

**SUPPLEMEMTARY APPENDIX A: Psychometric Scales**

| **Scales completed by the patient, parent and partner groups** | | | |
| --- | --- | --- | --- |
| **Scales** | **Description** | **Measure** | **Comparison sample from validation studies** |
| **EQ-5D-5L** | A measure of self-perceived health using a descriptive system and a visual analogue scale (EQ VAS). The descriptive system assesses 5 subscales of health including mobility, self-care, usual activities, pain or discomfort and anxiety or depression. | The subscales are rated on a 5-point scale and converted into a single index ‘utility’ score using an Australian value-set algorithm. The utility score ranges from 0 (dead) to 1 (full health). The EQ VAS measures self-rated health from 0 to 100, with higher scores indicating better health. | 9958 Australian adults that demographically represent the general population.^16^ |
| **Depression Anxiety and Stress Scale (DASS-21)** | A 21-item self-report measure assessing the degree of emotional distress across three domains: depression, anxiety and stress. | It is scored on a 4-point severity/frequency scale. Item scores within each domain are summed and compared against recommended cutoff values to indicate severity, ranging from normal (lower scores) to extremely severe (higher scores). | 785 Australian adults that demographically represent the census statistics.^17^ |
| **Rosenberg Self-Esteem Scale (RSES)** | A measure that assesses sense of self-worth. | It uses a 4-point Likert scale. Higher scores indicate higher self-esteem. Scores ≥25 indicate positive self-evaluation. | 485 Australian college students and community members.^19^ |
| **Emotion Thermometers (ET)** | A screening tool for emotional distress consisting of 5 visual-analogues scales, assessing distress, anxiety, depression, anger, and need for help. | Each scale is rated between 0 and 10. Higher scores indicate higher emotional distress. Scores ≥4.0 suggest clinically relevant distress. | 1362 German female adults representing the general population.^18^ |
| **Additional scales completed by patients** | | | |
| **Centrality of Event Scale (CES)** | A seven-item measure of the extent to which a stressful event has shaped an individual’s identity. | It is rated on a 5-point Likert scale. Higher scores indicate a stronger perceived connection between the event and current experiences and are correlating with the presence of post-traumatic stress disorder symptoms. | 707 North American undergraduate students.^21^ |
| **Fertility Quality of Life (FertiQoL)** | A measure of fertility-specific quality of life. Four core subscales including emotional (psychological health), mind-body (physical health), relational (partnership) and social (social interactions) were assessed. | The subscales and total scores are formulated to a score between 0 and 100, where higher scores indicate better fertility-related quality of life. | 1414 men and women experiencing fertility difficulties in Australia, Canada, New Zealand, UK and USA.^12^ |
| **Female Sexual Function Index (FSFI)** | A 19-item scale that assesses 6 domains of female sexual function over the preceding four weeks: desire, arousal, lubrication, orgasm, satisfaction, and pain. | Full-scale scores range from 2.0 to 36.0 with higher scores reflecting better sexual function. A total score above 26.55 indicates the absence of sexual dysfunction.^22^ | 244 women without sexual dysfunctions.^22^ |
| **Additional scales completed by patients and parents** | | | |
| **Family Assessment Device (FAD)** | A 12-item measure of perceived family functioning. | It uses a 4-point Likert scale. Higher scores indicate poorer family functioning. Scores ≥2.0 suggests problematic family function. | 1959 Western Australian primary caregivers. ^23^ |
| **Scales completed by patients in a relationship and their partners** | | | |
| **Relationship Assessment Scale (RAS)** | A seven-item scale assessing relationship satisfaction. | It consists of a 5-point Likert scale. High scores suggest better relationship function with scores above 4.0 signifying satisfactory partner relationship. | 149 North American women who were in relationships.^20^ |
